# The application of label-free Raman microscopy to monitor particle-cell interactions in *in vitro* experiments

**DOI:** 10.1016/j.csbj.2025.06.002

**Published:** 2025-06-04

**Authors:** Manosij Ghosh, Steven Ronsmans, Peter HM Hoet

**Affiliations:** Environment and Health, Department of Public Health and Primary Care, KU Leuven, Herestraat 49, Leuven 3000, Belgium

**Keywords:** Nanotoxicology, TiO2, Raman microscopy, Particle uptake, Microscopy

## Abstract

Characterizing particle–cell interactions is a critical component of nanotoxicology research, complementing material characterization to better interpret observed biological effects and explore dose–response relationships. However, accurate assessment of particle uptake remains challenging due to limitations in distinguishing internalized particles from those bound to the cell surface, labor-intensive sample preparation, and constraints in quantification methods. To address these challenges, label-free techniques with minimal sample processing are being explored. In this study, we present findings from a series of experiments using confocal Raman microscopy as a non-destructive, label-free method to evaluate particle–cell interactions. Suspensions of anatase (NM-102; 21 ± 10 nm) and rutile (NM-104; 26 ± 10 nm) TiO₂ nanoparticles were analyzed in two in vitro models: THP-1 (suspension) and 16HBE14o− (adherent) cells. Raman spectral analysis enabled the detection of cell-associated particles based on their unique chemical fingerprints. We further observed concentration-dependent trends in particle–cell interaction using semi-quantitative metrics such as particle area and spectral match. Lastly, optimal scan parameters were identified to improve detection efficiency while preserving simple sample preparation workflows.

## 1 Introduction

Characterizing nanoparticle–cell interactions in nanotoxicological studies, particularly in vitro, remains challenging due to complex sample preparation protocols, variability in method sensitivity, and the absence of standardized procedures. These limitations have been critically reviewed by Asraf et al.[1]

Techniques such as transmission electron microscopy (TEM) are commonly used to visualize particle localization and internalization, but they are not inherently quantitative and require labor-intensive sample processing. Other commonly used methods, including flow cytometry[2], [3] and inductively coupled plasma mass spectrometry (ICP-MS) or optical emission spectrometry (ICP-OES), can quantify relative concentration or total uptake, but do not distinguish between internalized and surface-adherent particles. These methods are limited by their use of live cells (flow cytometry) and/or have extensive sample preparation requirements (TEM, ICP-MS/OES). Additionally, some methods such as the ICP-MS and laser ablation ICP-MS (LA-ICP-MS) are inherently destructive.

In recent years, several label-free approaches have emerged as potential alternatives. Methods such as femtosecond pulsed laser scanning microscopy have emerged as promising alternatives [4], [5], [6], [7]. Other methods such as the confocal Raman microscopy also hold promise for non-destructive characterization and chemical mapping of fixed and live cells[8], [9].

In this context, we designed a simplified experimental workflow to explore the feasibility of using confocal Raman microscopy for characterizing nanoparticle–cell interactions, with potential applications in nanotoxicology. While not capable of distinguishing between internalized and surface-bound particles, Raman microscopy offers label-free detection with chemical specificity.

## 2 Materials and methods

### 2.1 Study design

**Fig. 1:**
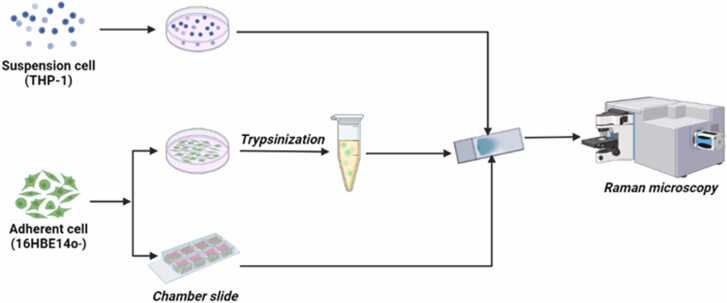
Schematic overview of the experimental design used for the present study; Created in BioRender

The general experimental protocol is represented in Fig. 1. Human monocyte (THP-1) cells were used as a representative of a suspension cell type, and were cultured in RPMI 1640 4 [supplemented with 10 % fetal bovine serum (FBS), 100 U/ml penicillin, 2 mM L-glutamine and 2.5 µg/ml fungizone][11]. Human bronchial epithelial cell line (16HBE14o-) was cultured in DMEM 5 [supplemented with 5 % FBS, 100 U/ml penicillin, 2 mM L-glutamine and 2.5 µg/ml fungizone][12] and served as representative of adherent cells in the present experiment. Cells were cultured at 37 °C in a 100 % humidified atmosphere containing 5 % CO_2_. Cell culture medium was renewed every 2–3 days and sub-cultured at confluence. For both cell lines, cells from passage 4–8 were used for all the experiments.

Two forms of TiO_2_-nanoparticles: anatase (NM-102; 21 ± 10 nm)[12] and rutile (NM-104; 26 ± 10 nm)[12], obtained from Joint Research Centre (JRC), were used. Stock suspension of each nanoparticle (2.5 mg/ml) was prepared by dispersing the powder in 2 % serum-water using probe sonication as previously described[12]. Cells were exposed to the freshly prepared concentrations of particles (2.5 and 25 μg/ml) for 24 h, before being processed for imaging as described below.

Comparison of the different cell types (suspension- THP1 vs adherent- 16HBE14o-) and comparison of processing methods of adherent samples (16HBE14o- trypsinized vs 16HBE14o- chamber slides) constituted Experiment 1. After exposure, THP-1 cells were washed three times with HBSS 6 and immobilized onto microscope slides using CytoSpin (Shandon, Cytospin 3) and air dried before being processed for imaging. 16HBE14o- cells, on the other hand, were exposed to particles and processed using two different methods for the imaging experiment. In the first method, cells were cultured and exposed directly in a chamber slide, post exposure cells were washed three times with HBSS, air dried and processed for imaging. In the second method, the 16HBE14o- cells were cultured and exposed in 24 well plates, following which the cells were enzymatically released using 0.1 % trypsin-EDTA (diluted 1/10 in HBSS), washed three times with HBSS and subsequently immobilized onto microscope slides using CytoSpin. The air-dried slides were imaged using the Thermo Scientific™ DXR3xi Raman Imaging Microscope, equipped with 532 nm laser. The collected spectral data were analyzed using the OMNIC™ Series software and built-in particle analyzer was used for particle detection. TiO_2_ polymorphs exhibit distinct Raman spectra which were used for qualitative and quantitative analysis. Experiment 2 on the other hand constituted of an independent experimental setup using 16HBE14o- (chamber slides) to further refine the scan settings to obtain high quality spectral data. Different scan settings used in the experiments are summarized in Table 1.

**Table 1:** Summary table of different experimental setup used in the present study.

Experiment	Description	Scan setting
Experiment 1	THP−1 16HBE14o- (trypsinized) 16HBE14o- (chamber slides)	Laser frequency: 532 nm Laser power: 2 mW Exposure time: 0.01 sec (100 Hz) Number of scans: 3 Image pixel size: 0.3 µm Aperture: Pinhole 50 micron
Experiment 2	16HBE14o- (chamber slides) scan setting- 3 Scans & 0.5 µm- 3 Scans & 0.3 µm- 5 Scans & 0.5 µm- 5 Scans & 0.3 µm	Laser frequency: 532 nm Laser power: 2 mW Exposure time: 0.02 sec (50 Hz) Number of scans: 3 or 5 Image pixel size: 0.3 µm or 0.5 µm Aperture: Pinhole 50 micron

## 3 Results and discussion

**Fig. 2:**
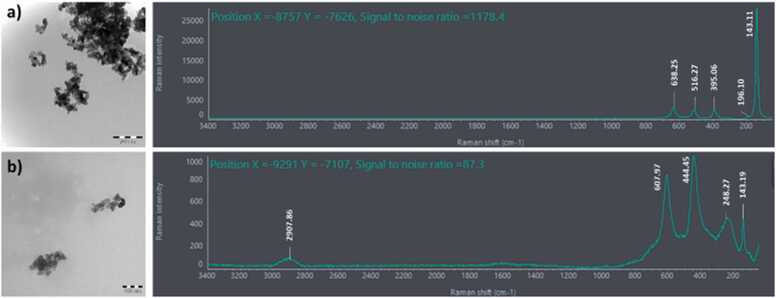
TEM images (collected as part of previous experiment)[12] and corresponding Raman spectra for a) anatase -NM-102, and b) rutile- NM-104 TiO_2_-nanoparticles. In the graphical depiction of Raman spectra, the intensity of the scattered light is plotted on the y-axis and the frequency of light (Raman shift, wavenumber cm^−1^) is on the x-axis.

At the onset, reference Raman spectra were acquired from the JRC reference materials (NM-102 and NM-104). The anatase particle (NM-102, Fig. 2a) exhibited major peaks at 638, 516, 395, 196 and an intense peak at 143 cm^−1^, while the rutile phase (NM-104, Fig. 2b) had major peaks at approximately 608, 444, 248 and 143 cm^−1^. The spectral information collected was comparable to that described earlier[13].

**Fig. 3:**
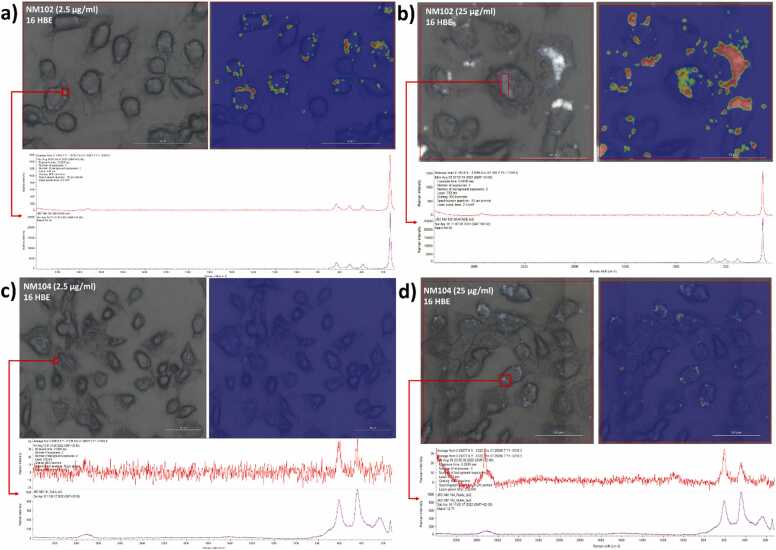
Qualitative detection of particles in 16HBE14o- cells exposed to a) NM-102 (2.5 µg/ml) b) NM-102 (25 µg/ml), c) NM-104 (2.5 µg/ml) and d) NM-104 (25 µg/ml); blue images in inset with red/orange dots are representative of identified particle using the particle analyzer of OMNIC™ series software. Upper spectra (red) represent the scanned region; lower spectra (purple) show the matched library reference.

Once the reference spectra were acquired, the first step was to qualitatively check if it was possible to accurately identify particles associated with cells. 16HBE14o- cells exposed to NM-102 (2.5 and 25 µg/ml) and NM-104 (2.5 and 25 µg/ml) were qualitatively evaluated, following which particle spectra were matched to spectra obtained from the reference materials for confirmation (Fig. 3). Once the spectra of the particles associated with the cells were confirmed to be that in the library, additional experiments were performed to quantify the number and size of the particles and understand the influence of different sample preparation protocols on particle detection.

As a next step in the optimization procedure, experiments were setup towards semi-quantitative estimation of particle interaction with cells (THP-1 and 16HBE14o-, Table 1- experiment 1). As part of the experiment, a minimum of 40 regions was scanned per condition to collect spectral information, each region representing one cell. The collected spectral data were analyzed using the OMNIC™ Series software and built-in particle analyzer application. This provided i) confirmation on the chemistry of the particles based on library matching, ii) number of cells in contact with particles and iii) an estimate of the number of particles per cell.

In the first experiment (Table 1- experiment 1), for the three cell processing conditions used, namely i) THP-1, ii) 16HBE14o- (trypsinized) and iii) 16HBE14o- (chamber slides), particles were accurately identified. In the scanned regions the smallest area covered by the particle was 0.8 µm^2^ (which corresponds to approximately 1 µm in diameter, assuming spherical particle aggregates). Larger aggregates of particles (> 35 µm^2^) were also present at the higher exposure conditions (25 µg/ml), but no conclusion could be drawn regarding total particle concentration or dose-response relationships.

It is important to mention here that the method used has limited ability to distinguish between surface-bound and internalized particles. Therefore observations are interpreted as “particle-cell interactions” rather than “particle uptake”, aligning with the scope of confocal Raman microscopy.

**Fig. 4:**
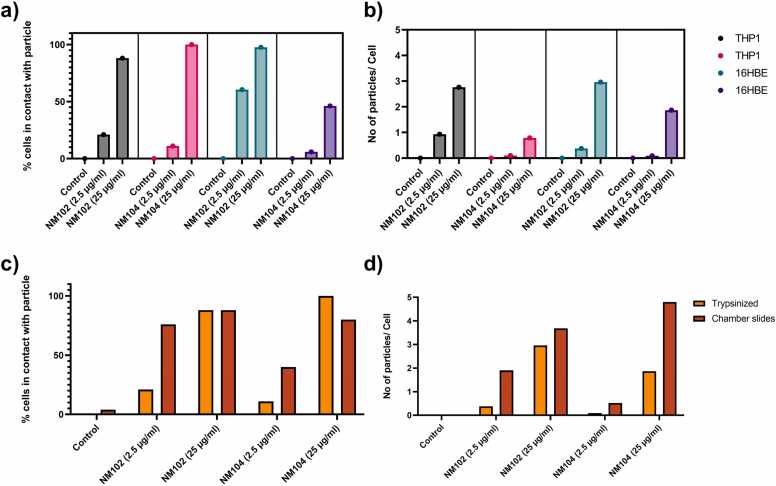
Comparison of particle-cell interaction, [a and b] between THP-1 and 16HBE14o- (trypsinized) cells, and [c and d] between 16HBE14o- (trypsinized) and 16HBE14o- (chamber slides) setup, exposure conditions NM-102 (2.5 µg/ml and 25 µg/ml), NM-104 (2.5 and 25 µg/ml). Results are expressed as % cells in contact with particles and number of particles/ cell.

While concentration-dependent cell-particle interactions were observed for NM-102 and NM-104, no significant differences were found between THP-1 and 16HBE14o- (trypsinized) in terms of % cells in contact with particles or number of particles/ cell (Figs. 4a and 4b). However, sample handling influenced the results significantly, as trypsinized 16HBE14o- cells showed fewer particle interaction compared to adherent 16HBE14o- cells (chamber slides, Figs. 4c and 4d). This reduction may be attributed to particle detachment during trypsinization, suggesting that chamber slide preparation better retains membrane-bound particles.

**Fig. 5:**
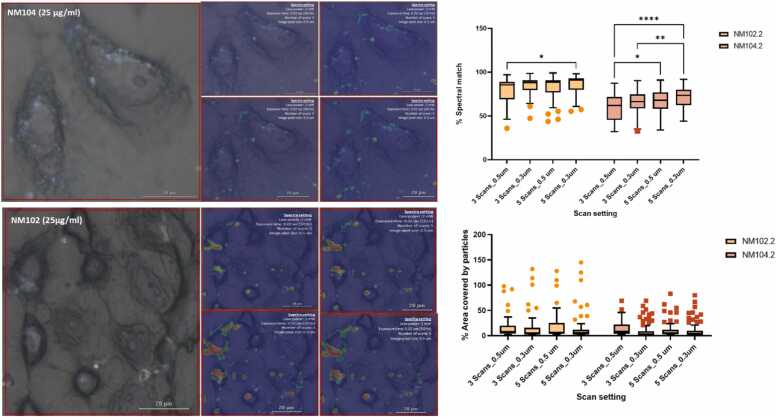
Representative fields showing comparison of different scan setting on spectral quality in 16HBE14o- (chamber slides) setup exposed to NM-102 (25 µg/ml), NM-104 (25 µg/ml), results are expressed as % area covered by particles and % spectral match. * p < 0.05, ** p < 0.001, **** p < 0.0001- two-way ANOVA-Tukey's multiple comparisons test.

To refine the scanning protocol, 16HBE14o- adherent cells (chamber slides) exposed to 25 µg/ml of NM-102 and NM-104 were scanned using different settings (Table 1- experiment 2). Three representative regions per condition were scanned using combinations of scan counts and image pixel size (3 scans & 0.5 µm, 3 scans & 0.3 µm, 5 scans & 0.5 µm, 5 scans & 0.3 µm). While area coverage by particles remained consistent, the setting of 5 scans with 0.3 µm pixel size produced the highest spectral match percentages for both particle types (Fig. 5). The improvement was more pronounced for rutile (NM-104), likely due to its lower Raman scattering intensity compared to anatase.

Based on the results of the present study and previous work utilizing confocal Raman microscopy for characterizing particles in both cellular and cell-free systems[14], [15], [16], [17], we conclude that the method can be applied effectively in nanotoxicology. While the current setup does not enable mapping of intracellular distribution or uptake, it enables reliable detection and relative quantification of particle-cell interactions using a simple, non-destructive protocol.

Compared to other optical methods such as hyperspectral imaging with enhanced darkfield microscopy (HSI-M), which has been used to detect and semi-quantify various metal-based nanoparticles, including TiO₂, in biological systems [10], Raman spectroscopy offers distinct advantages. While HSI-M allows for rapid, label-free visualization of nanoparticles and can differentiate between types based on spectral profiles, it lacks the chemical fingerprinting capability inherent to Raman spectroscopy. Therefore, Raman spectroscopy provides complementary benefits, particularly for confirming particle identity in complex biological matrices. Additionally, Raman spectroscopy has a strong foundation in the field of drug delivery, where it has been used to monitor intracellular fate and subcellular localization of nanocarriers and bioactive compounds. As summarized in a recent review by Liu et al. [18], its application in nanoparticle tracking has been well established, further supporting the feasibility of extending Raman methods for nanotoxicological investigations.

The complexity of the detection method may be further enhanced to explore subcellular localization of particles, by leveraging Raman spectra of different cellular components[19] or combining with complementary imaging techniques. However, depending on the study design and materials involved, additional optimization and spectral library development may be necessary.

In conclusion, although mapping intracellular particle distribution or confirming true internalization cannot be achieved using the current label-free Raman microscopy approach, the method provides a reliable and chemically specific means to detect particles and assess relative particle-cell interactions. With further development and integration with complementary techniques, this method may be extended for advanced applications in both nanotoxicology and nanomedicine.

## CRediT authorship contribution statement

**Manosij Ghosh:** Writing – review & editing, Writing – original draft, Visualization, Software, Resources, Methodology, Investigation, Formal analysis, Data curation, Conceptualization. **Steven Ronsmans:** Writing – review & editing, Methodology. **Peter HM Hoet:** Writing – review & editing, Supervision.

## Declaration of Competing Interest

The authors declare that they have no known competing financial interests or personal relationships that could have appeared to influence the work reported in this paper.

## Data Availability

The data supporting the findings of this study are available in Supplementary Files. Large raw data files (e.g.,.MAPX Raman scans) are available on request or will be deposited in the KU Leuven repository. A DOI will be provided upon acceptance.

## Footnotes

4 Roswell Park Memorial Institute (RPMI) 1640 Medium

5 Dulbecco's Modified Eagle Medium

6 Hanks’ Balanced Salt solution
